# SOGUG Multidisciplinary Expert Panel Consensus on Updated Diagnosis and Characterization of Prostate Cancer Patients

**DOI:** 10.3390/curroncol33010061

**Published:** 2026-01-20

**Authors:** Enrique Gallardo, Alfonso Gómez-de-Iturriaga, Jesús Muñoz-Rodríguez, Isabel Chirivella-González, Enrique González-Billababeita, Claudio Martínez-Ballesteros, María José Méndez-Vidal, Mercedes Mitjavila-Casanovas, Paula Pelechano Gómez, Aránzazu González-del-Alba, Fernando López-Campos

**Affiliations:** 1 Department of Oncology, Parc Taulí Hospital Universitari, Institut d’Investigació i Innovació Parc Taulí (I3PT-CERCA), Universitat Autònoma de Barcelona, 08202 Sabadell, Spain; 2 Hospital Universitario de Cruces, Vizcaya, Biobizkaia Health Research Institute, Basque Country University (UPV/EHU), 48940 Santsoena, Spain; 3 Department of Urology, Parc Taulí Hospital Universitari, Institut d’Investigació i Innovació Parc Taulí (I3PT-CERCA), Universitat Autònoma de Barcelona, 08202 Sabadell, Spain; 4 Servicio Oncología Médica, Hospital Clínico Universitario, INCLIVA, Universidad de Valencia, 46010 Valencia, Spain; 5 Hospital General Universitario 12 de Octubre, 28041 Madrid, Spain; 6 Hospital Universitario Puerta de Hierro-Majadahonda, 28222 Madrid, Spain; 7 Medical Oncology Department, Maimonides Institute for Biomedical Research of Córdoba (IMIBIC), Hospital Universitario Reina Sofía (HURS), 14004 Córdoba, Spain; 8 JSº Medicina Nuclear, Hospital Universitario Puerta de Hierro-Majadahonda, 28222 Madrid, Spain; 9 Servicio de Radiología, Fundación Instituto Valenciano de Oncología (IVO), 46009 Valencia, Spain; 10 Department of Medical Oncology, Hospital Universitario Puerta de Hierro-Majadahonda, 28222 Madrid, Spain; 11 Radiation Oncology Department, Hospital Universitario Ramón y Cajal, Madrid, Genesis Care Hospital Vithas La Milagrosa, 28010 Madrid, Spain

**Keywords:** prostate cancer, screening, multidisciplinary units, next-generation imaging, germline genetic alterations, molecular biomarkers

## Abstract

A group of experts of different specialties involved in the care of prostate cancer (PCa) patients participated in the ENFOCA2 project, promoted by the Spanish Oncology Genitourinary Group (SOGUG), with the aim to review, discuss, and summarize current relevant aspects related to screening, diagnosis, imaging, risk-based approach, and molecular characterization of PCa. A multidisciplinary team (MDT) approach is essential to ensure that patients receive evidence-based care, promoting shared decision-making, and tailoring treatment to the patient’s unique values and preferences. Population-based screening based on risk-stratified algorithms is needed to overcome the limitations of opportunistic screening for detecting clinically significant PCa. Next-generation imaging (NGI) methods, such as prostate-specific membrane antigen (PSMA) PET/CT alone or combined with multiparametric MRI (mpMRI), have a promising role in different scenarios of the diagnostic process due to their high sensitivity. The diagnostic yield of mpMRI should be improved, especially for assessing extraprostatic extension. The use of specific molecular probes as imaging markers for MRI could improve the staging of metastatic disease. Protocols for germline testing developed by international societies, such as the European Association of Urology (EAU) and the National Comprehensive Cancer Network (NCCN), should be adapted at local levels, with *BRCA1/2*, *ATM*, *PALB2*, *CHEK2*, *MLH1*, *MSH2*, *MSH6*, *PMS2*, *EPCAM*, and *HOXB13* as the genes to be investigated. Genomic classifier tools help identifying aggressiveness of cancers and aid in personalized treatment decision-making. Joint efforts of multidisciplinary physicians are crucial to improve health outcomes for patients with PCa across the spectrum of this disease.

## Simple Summary

A group of experts in the care of patients with prostate cancer analyzed challenging aspects related to the diagnosis and management of the disease. These specialists reviewed evidence-based data reported in the literature, and after discussion together with their own experience, a narrative review was developed. The aim of the review was to provide updated information on diagnostic aspects and the most adequate treatment approaches for different stages of prostate cancer. Some important conclusions included: (1) the need to provide care within the framework of a multidisciplinary team; (2) the importance of tailoring treatment to individual preferences and values in a shared decision-making model, particularly regarding screening of prostate cancer; (3) the promising role of imaging techniques, such as prostate-specific membrane antigen/positron emission tomography/computed tomography due to their sensitivity; and (4) to focus further research in validation studies of molecular biomarkers for improving characterization of prostate cancer in clinical practice.

## 1. Introduction

Prostate cancer (PCa) is the most frequent cancer in men and ranks fourth with regard to mortality according to the latest GLOBOCAN 2022 statistics [1]. Despite impressive advances in the understanding of molecular mechanisms and complex tumor biology of PCa, rapidly evolving diagnostic and treatment landscapes have led to several challenges and controversies on ways that clinicians and multidisciplinary teams can address different scenarios across the cancer disease continuum, particularly given the accelerating advances in targeted therapy and precision medicine [2].

The ENFOCA2 project, promoted by the Spanish Oncology Genitourinary Group (SOGUG), was designed as a space for the integration of perspectives of a panel of different Spanish specialists involved in the management of PCa patients, with the aim of providing updated information on relevant and clinically applicable aspects for optimal oncological care. At the time of developing and reaching consensus on this information, evidence-based recommendations of leading authorities, such as the European Association of Urology (EAU) https://uroweb.org (accessed on 15 January 2026) and the National Comprehensive Cancer Network (NCCN) https://www.nccn.org (accessed on 15 January 2026), were also considered. The main topics to be discussed were selected by the expert panel and included the following: (1) development and application of multidisciplinary units from early stages of the disease; (2) usefulness of population-based screening; (3) indications and access to novel imaging diagnostic methods including next-generation imaging; (4) identification of patients with germline genetic alterations, genetic counselling, and recommendations for risk-adapted screening; and (5) clinical application and molecular and genetic characterization of the disease. For each topic, an overview of the current situation/state of the art is presented, followed by identification of demanding needs, and a proposal of recommendations to overcome these challenges. The final texts were discussed and agreed upon by members of the panel in various meetings.

## 2. Development and Application of Multidisciplinary Units from Early Stages of the Disease

### 2.1. Overview of the Current Situation

PCa is the most common malignancy in men in more than 100 countries, and a major cause of mortality and disability [1,2]. According to the Lancet commission on cancer of the prostate, by 2040, it has been estimated that the number of prostate cancer patients would be doubled to 2.9 million annually, with an 85% increase in annual deaths, although these figures will be higher due to missing data and underdiagnosis in middle- and low-income countries [3]. These latest statistics highlight the growing burden of PCa and the urgent need to adequately address the needs of cancer care for these patients.

In addition, PCa has long been recognized as a disease with a heterogeneous phenotype and significant inter-patient variability. Different treatment options may be indicated based on the specific characteristics and risk classification of patients [4], but the challenge lies in selecting the optimal therapeutic strategy to improve both the quality of life and the outcome for each individual case. The introduction of novel imaging technologies, such as prostate-specific membrane antigen (PSMA) PET/CT with ^18^F-DCFPyL or ^68^Ga-gozetotida, has the potential to modify staging and the indication of treatment [5,6,7,8]. Improved accessibility to genetic sequencing techniques for analyzing germline mutations in DNA damage repair genes may also assist in the selection of treatment in patients with metastatic disease [9,10,11]. The multimodal treatment approach and the rapid evolution in diagnostic and treatment landscapes, driven by advances in imaging and genetic testing, have increased the potential for precision medicine in PCa. However, clinicians frequently encounter difficulties due to insufficient knowledge on how to effectively integrate these advances into daily practice.

The biological and clinical heterogeneity of PCa with the risks of both undertreatment and overtreatment, the chronic and persistent disease course requiring individualized and sequencing decisions, and the availability of multimodal treatment options at each stage of the disease highlight the need for a multidisciplinary team (MDT) approach. MDTs are intended to ensure that patients receive evidence-based care, promote shared decision-making, and tailor treatment to a patient’s unique values and preferences. Moreover, MDT reduces bias and increases adherence to evidence-based guidelines, resulting in greater accuracy/efficiency of diagnostic methods and better clinical outcomes.

**Figure 1:**
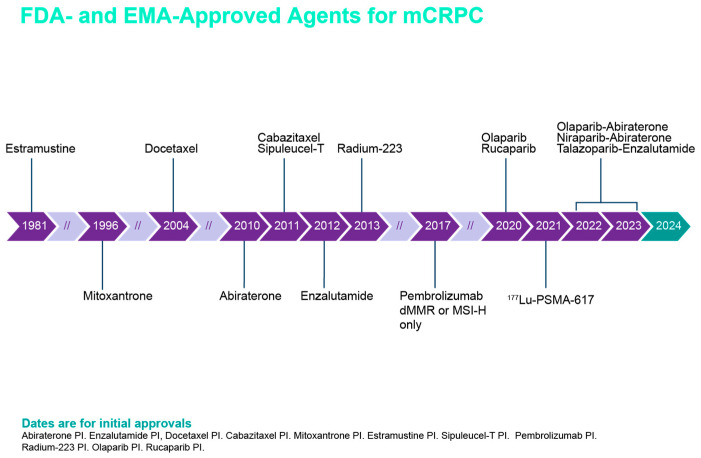
Different therapies that have been FDA-approved over the past years for the treatment of PCa patients with advanced disease.

New combined therapies of effective drugs are associated with longer survival rates, preserving the quality of life. Also, the treatment strategies can be adapted to the patients’ profiles and preferences because of the availability of different treatment alternatives. In this respect, MDT contributes to optimizing shared decision-making according to changing treatment strategies of PCa (Figure 1). This MDT approach requires the integration of different specialties to assess the clinical application of next-generation nuclear medicine imaging (NGI) techniques expected to reshape the diagnosis, staging, guidance of treatment, and monitoring of response [5], recommendations of germline and somatic genetic testing in several specific patients subgroups to inform prognosis/risk stratification and targeted treatment decisions (e.g., PARP inhibitors, pembrolizumab), and the opportunities offered by technologies based on artificial intelligence (AI) in the diagnostic, prognostic, and treatment fields [2]. In this respect, a new AI-derived predictive biomarker has been validated to assess the benefit of androgen-deprivation therapy (ADT) in men with intermediate- and high-risk localized PCa treated with radiotherapy [12].

Typical members of a PCa MDT include urologists, medical oncologists, radiation oncologists, nurses, and radiologists and nuclear medicine physicians and specialists, with additional participation of pathologists, genetic counselors, and molecular testing experts, who are increasingly playing prominent roles in current practice. MDTs play a crucial role during the entire spectrum of the disease (diagnosis, staging, treatment, follow-up, monitoring) as shown in Table 1.

The implementation of Prostate Cancer Units (PCU) was proposed by the European School of Oncology (ESO) in 2011 as the most adequate structures for the integration of MDT in the care of PCa patients at all stages of the disease, including prevention and treatment of complications as well as provision of psychological and emotional support related to the impact of prostate cancer and its treatments [13]. Other advantages of PCUs include reduced economic costs and avoidance of consultations with different specialists and inadequate therapies. It is concluded that European countries should consider the certification of PCU Units as a necessary way forward to ensure that men with PCa receive optimal treatment and care [13]. In 2012, the PCU *Initiative in Europe* was launched, and a multiprofessional Task Force gathered a set of standards for quality comprehensive care of PCa patients and designated pathways in PCUs [14]. A total of 40 standards/indicators and requirements/measurable elements were defined, which will have to be taken into account by institutions interested in setting up a PCU and future accreditation [14]. In 2019, and under the auspices of the European Association of Urology (EAU), experts on PCa diagnosis and treatment defined a set of criteria within the steps of clinical care, research, and education (including a quality control approach for the three steps), to identify the European Prostate Cancer Centers of Excellence (EPCCE) [15].

In addition, value-based healthcare models based on a multidisciplinary approach facilitate coordination of patients’ care and measurement of outcomes, with extended benefits to patients themselves, healthcare providers, suppliers, payers, and the community as a whole [16]. The International Consortium for Health Outcomes Measurement (ICHOM) has defined a standard set of patient-centered outcomes that should be measured in newly diagnosed men with localized PCa as a crucial first step in improving the value of care [17]. Currently, data on the quantitative impact of using MDTs in these modern scenarios are lacking, but MDTs play an essential role in creating standardized care pathways. However, it is necessary to evaluate benefits and costs, adherence to evidence-based guidelines, as well as financial costs, time to next treatment, overall survival (cure), freedom from biochemical failure, and patient-reported outcome metrics.

### 2.2. Challenges

- Convince specialists from all specialties involved in PCa of the convenience and need of a multidisciplinary approach, placing the patient at the heart of healthcare.
- Make multidisciplinary unit official and provide it with resources. To this purpose, it is essential to convince the decision-making bodies (management and health departments).
- Assume that decision-making must occur within the multidisciplinary unit and its committee and that it be binding.
- Protocolize decision-making.
- Incorporate new diagnostic methods in a protocolized and evidence-based approach.
- Effectively incorporate specialties: pharmacy, geriatrics, nursing/case management, basic/translational research, and the clinical trials unit.

### 2.3. Recommendations

- Publicize the interest in the formation of multidisciplinary units: monographic meetings, development dossier, course/diploma on the topic.
- Having data from existing multidisciplinary units available to demonstrate its usefulness.
- Give official status to the multidisciplinary unit, with a public and effective organizational body.
- Present all the information to decision makers to try to positively influence the implementation.
- Develop action-oriented protocols based on available scientific evidence.
- Establish joint consultations with urology, medical oncology, radiation therapy, and geriatrics (if possible).
- Involve pharmacy in decision-making.
- Add to the multidisciplinary unit the clinical trials and case management units.

## 3. Usefulness of Population-Based Screening

### 3.1. Overview of the Current Situation

The efficacy of using prostate-specific antigen (PSA) for screening of PCa was evaluated in two large studies, with inconsistent results. The Prostate, Lung, Colorectal, and Ovarian (PLCO) Cancer Screening Trial involved about 38,000 men in the intervention (annual PSA tests for 6 years and digital rectal examinations [DRE] for 4 years) and control arm, and after an extended follow-up over a median of 15 years, no benefit of the intervention in reducing mortality was found [18]. Because of the high rate of control-arm PSA testing, this finding can be viewed as showing no benefit of organized screening versus opportunistic screening. However, a 16-year report from the European Randomized Study of Screening for Prostate Cancer (ERSPC) (a multicenter population-based randomized screening trial with 182,160 men conducted in eight European countries) reported that a longer follow-up increased the absolute effect of PCa screening on mortality [19]. Among screened men, the PCa excess incidence decreased, but was still rather high. Reduction in PCa mortality seems to be related to screening duration, but a one-time screening test appeared to have no effect or little effect on the mortality of PCa because of a pool of prevalence of advanced disease in which relevant benefits from treatment are unlikely [19].

With the results derived from these two clinical trials and considering the large number of opportunistic screenings, an updated Council of the EU recommendation on PCa screening invited countries to proceed with a stepped approach based on piloting and further research to assess the feasibility and effectiveness of the implementation of organized screening programs, ensuring quality and appropriate management based on PSA testing followed by MRI scanning [20].

The US Preventive Services Task Force (USPSTF) recommendation on PSA-based screening for PCa reviewed the evidence and recommended that in men aged 55 to 69 years, the decision to be screened should be an individual one, whereas men 70 years or older should not undergo screening [21]. Moreover, specific recommendations for PSA-based screening in men with a family history of PCa were not made by the USPSTF according to the available evidence. Potential benefits may be offered by PSA screening to these patients as compared with the general population, but there is the potential to increase exposure to harms, particularly in men with cancer overdiagnosis in their relatives. It is probable that men who would mostly benefit from screening would be those with a first-degree relative who had an advanced cancer at initial diagnosis, presented metastatic disease, or died from PCa. Also, screening may be more beneficial in African American men versus the general population, but current evidence supporting this statement is lacking [21].

The recommendations of other societies are diverse. The position of the American Academy of Family Physicians, in 2018, was to not routinely screen for PCa using a PSA test or DRE [22]. The Canadian Task Force on Preventive Health Care (CTFPHC), in 2014, recommended not screening for PCa in men aged less than 55 years and 70 years of age or older, as well as in men aged 55–69 years, although the risks and benefits of PSA screening and its potential consequences should be discussed with each patient in the context of his preferences [23]. The American College of Physicians (ACP), in 2013, reported similar recommendations with no PSA screening in men under the age of 50 years or over the age of 69 years, whereas clinicians should inform men between the ages of 50 and 69 years about the limited potential benefits and substantial harms of PCa screening [24]. The American Urology Association (AUA), in 2023, recommended that clinicians should offer a baseline PSA test to people between ages 45 to 50 years, cancer screening beginning at age 40 to 45 years for people at increased risk (Black ancestry, germline mutations, strong family history of PCa), and regular prostate cancer screening every 2 to 4 years to people aged 50 to 69 years [25]. The European Association of Urology (EAU), in 2024, summarized guidelines based on the strength of recommendation, but also recognizing that population or mass screening for PCa remains one of the most controversial topics in the urological literature. A summary of the EAU recommendations [26] is shown in Table 2.

In summary, based on data from the aforementioned studies and recommendations of different societies, population-based screening programs have not demonstrated cost-effectiveness, and their generalized use does not appear to be recommended. In contrast, opportunistic PCa screening can be considered, relying on a shared decision-making approach.

In relation to the best method of risk-based PCa screening before biopsy, flexible algorithms according to local resource availability have been proposed, but the essence of all of them is to break the link between elevated PSA and immediate biopsy to reduce unnecessary biopsy procedures and overdiagnosis [27].

In 2021, Van Poppel et al. [28] published another algorithm for early diagnosis of PCa in a population-based setting that emerged from expert consensus, starting in well-informed men with PSA interval tests, followed by the selection of patients for imaging studies and biopsy based on calculators of PCa risk. Then, prostate MRI is offered to patients classified into intermediate and high risk. The combined results from risk calculators and MRI results can be used to select men for prostate biopsy. Then, all subsequent low-risk or biopsy-negative men will be offered a safety net consisting of PSA interval tests and repeated MRI if suspicion of clinically significant PCa persists. The authors also presented actions to be taken by age group (50–59 and 60–70) and PSA levels (<1, 1–3, and ≥3 ng/mL) [28]. One year later, this algorithm was further adapted [29]. In 2022, a Swedish group designed and implemented organized prostate cancer testing based on a stratified risk diagnostic algorithm according to results of PSA, PSA density, MRI data, and age [30]. Prostate biopsy is indicated in the presence of PSA ≥ 3 ng/mL, an MRI suspicious lesion, and/or PSA density ≥ 0.15 ng/mL/cm^3^.

**Figure 2:**
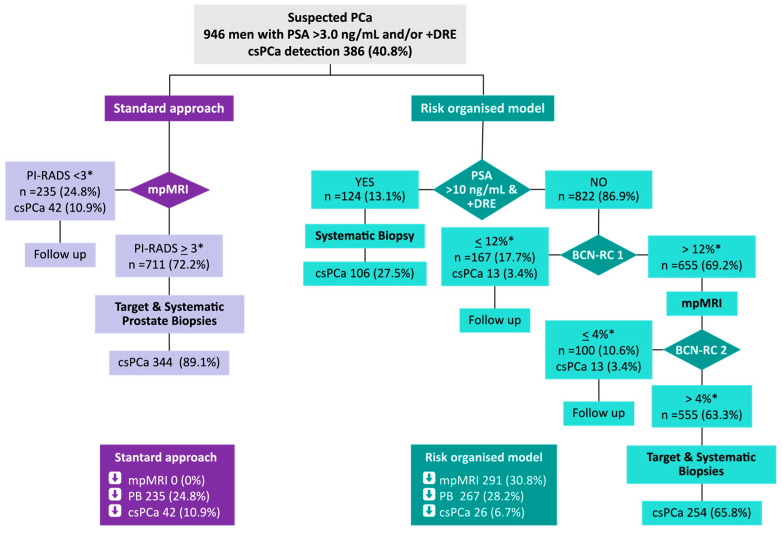
Comparison of the standard approach of clinically significant PCa (csPCa) and the proposed risk-organized model (ROM) (DRE: digital rectal examination; PI-RADS: prostate imaging reporting system; mpMRI: multiparametric magnetic resonance imaging; BCN: Barcelona; PB: prostate biopsies). * Proposed thresholds. [31].

In 2023, Morote et al. [31] used a risk-organized model (ROM) to improve early detection of clinically significant PCa (csPCa) by reducing the demand for unnecessary biopsies of the gland and multiparametric MRI (mpMRI) studies. In a group of 946 men with PSA > 3.0 ng/mL and/or positive DRE, the use of ROM was able to exclude almost one-third of mpMRI examinations, and the percentage of saved biopsies increased from the 24.8% observed with the standard approach to the 28.2%, whereas the percentage of undetected csPCa decreased from 10.9% to 6.7%, respectively. The comparison of the standard approach of csPCa and the proposed ROM is shown in Figure 2.

### 3.2. Challenges

- Implement population-based organized screening programs on a national level.
- Decrease the side-effects of early detection protocols, leading to substantial overdiagnosis and resulting in overtreatment.
- Better identification of individuals at risk.
- Incorporation of the latest knowledge and technology in risk-adapted screening programs.
- To overcome the practice of DRE-only-based screening or one-time PSA testing in the core age group of 55–70 years.
- Better definition of low-risk and high-risk groups to allow intensive and less intensive screening for improving cost-effectiveness.

### 3.3. Recommendations

- Evaluation of the usefulness of the development and implementation of a population-based PCa screening program (and not only opportunistic screening) from the perspective of medical scientific societies, healthcare organizations, and governmental bodies.
- It is necessary to elaborate a PCa screening algorithm agreed upon by the different medical societies, healthcare organizations, and local and national governmental departments, including the Ministry of Health.
- Assessment of the cost-effectiveness of the proposed algorithms, given that they will include a large population segment.
- It is necessary to develop strategies for the progression of algorithms in age groups and/or populations at higher risk.
- The beginning of population-based screening must be accompanied by the provision of financial resources since it will be associated with an increase in outpatient consultations, complementary examinations, and biopsies.
- It would be advisable to start with a pilot experience in a restricted age group and evaluate its results before extending it to the overall target population.

## 4. Indications and Access to Novel Imaging Diagnostic Methods

### 4.1. Overview of the Current Situation

Prostate MRI has shown a high sensitivity and high negative predictive value for the diagnosis of csPCa. The integration of MRI in the diagnostic pathway of subjects with suspicion of PCa has shown to be associated with significant benefits, including a decrease in the number of subjects undergoing prostate biopsy, a decrease in the detection of clinically insignificant PCa, and an increase in the detection of csPCa [32]. The role of MRI in the current diagnostic approach of PCa includes different aspects: (a) it is necessary to perform MRI before prostate biopsy [26]; (b) MRI studies are important to take the decision of whether or not prostate biopsy should be indicated according to Prostate Imaging Reporting System (PI-RADS) category [33] and PSA density (PSAD); (c) MRI/ultrasound fusion increases the yield of the biopsy procedure [34]; (d) is the technique of choice for local staging (T category); and (e) provides key information for treatment planning as well as for the follow-up of patients (identification of local recurrence, active surveillance, focal treatment).

Accurate local staging is critical for treatment planning and prognosis in patients with PCa, with the primary aim to differentiate between organ-confined and locally advanced disease. Multiparametric MRI (mpMRI) is a gold-standard imaging method providing high-quality images with excellent tissue contrast for identification of T2 staging (organ confined), T3a (extraprostatic extension), T3b (seminal vesicles invasion), and T4 (spread into other organs nearby). A systematic review and meta-analysis of 17 studies with a total of 3602 men analyzed the diagnostic usefulness of six MRI features of the PI-RADS for predicting extraprostatic extension (EPE) of the disease [35]. Two signs, including breach of the capsule with direct tumor extension and tumor-capsule interface >10 mm, were associated with the highest pooled specificity (98%) and sensitivity (86.3%), as well as the highest diagnostic odds ratio (OR) of 15.6 and 10.5, respectively.

MRI findings are critical for the assessment of EPE and neuro-vascular bundle preservation at the time of surgical planning, definition of treating volumes and neighboring organs for radiation therapy, detection of local recurrence after radical prostatectomy or radiotherapy/brachytherapy, and during active surveillance.

In relation to imaging diagnostic methods for the detection of lymph node and distant metastases, conventional computed tomography (CT) has a limited value for the assessment of nodal metastasis, as the size of nodes is not a sufficient criterion. CT is highly sensitive for osteolytic and osteoplastic bone lesions involving cortical bone, but less so for tumors restricted to the marrow space, which must be very extensive to be detectable [36]. MRI of the axial skeleton (AS-MRI) improves the detection of bone disease as compared with CT and ^99m^Tc bone scintigraphy [37,38]. In a study that evaluated bone metastasis in 66 patients with high-risk PCa, AS-MRI showed 100% sensitivity and 88% specificity as compared with 46% and 32% for ^99m^Tc bone scintigraphy alone [39]. Whole-body diffusion-weighted MRI (WB-DWI), a next-generation imaging (NGI), can identify metastatic bone disease with greater sensitivity than conventional imaging and is feasible for the detection of nodal and visceral metastasis in advanced PCa [40,41]. On the other hand, the Metastasis Reporting and Data System for Prostate Cancer represents the consensus recommendations on the performance, quality standards, and reporting of WB-MRI for use in all oncologic manifestations of advanced prostate cancer [42].

Next-generation imaging (NGI), such as PSMA PET/CT, provides higher diagnostic accuracy in primary staging of intermediate-to-high risk PCa patients as compared to standard CT. In a systematic review and meta-analysis of 31 studies, including 2431 patients, head-to-head comparison of PSMA PET/CT was more sensitive than mpMRI for detection of extra-prostatic extension, more sensitive and specific than mpMRI for nodal staging, and more sensitive and specific than BS with or without SPECT for bone metastasis staging [43]. These results suggest that PSMA PET/CT may be used as a first-line approach for the initial staging of PCa. Also, the combination of MRI + PSMA reduces false negatives for csPCa compared with MRI, potentially allowing a reduction in the number of prostate biopsies required to diagnose csPCa [44].

Although PSMA PET/CT has shown a promising role for the initial staging, mpMRI is the gold standard for local T-staging of PCa [45]. Extended pelvic nodal dissection (ePLND) at the time of radical prostatectomy remains the gold standard for the detection of lymph node invasion. Micrometastases are not detected by imaging techniques, and in order to limit the number of unnecessary lymphadenectomies, it has been recommended to perform ePLND according to the currently available nomograms (Briganti 2019 nomogram) [46]. PSMA PET/CT may be used to rule out nodal involvement in low-risk PCa patients, but, conversely, in high-risk patients, negative PSMA PET/CT cannot allow avoiding PLND [47]. In a cohort of 50 patients with intermediate/high risk PCa, PSMA PET/CT as preoperative staging examination, showed low sensitivity in detecting lymph node metastasis (25%), high specificity in excluding lymph-node disease (81%), and a positive correlation between lymph node standardized uptake value (SUV) and prostate involvement, suggesting that PSMA PET/CT could reflect the pathological features of the prostate [48]. Regarding the impact of imaging findings on change of treatment, data from the proPSMA study showed a greater change of treatment modality, delivery, or treatment intent in the PSMA PET/CT arm as compared with the conventional imaging arm (28% vs. 15%) [5]. On the other hand, in patients scheduled for radical prostatectomy based on conventional imaging (*n* = 53) or PSMA PET/CT (*n* = 53), the rates of free surgical margins and PSA persistence were similar, but in a subanalysis of high-risk patients, PSA persistence was significantly lower in the PSMA PET/CT group [49]. Finally, the use of specific molecular probes as imaging markers for MRI could improve staging of metastatic disease [50].

Although the available evidence from pivotal trials is based on conventional imaging (CT, bone scintigraphy), the incorporation of PSMA-based PET/CT into routine clinical practice requires clinicians and researchers to make decisions based on new information and on the earlier diagnosis of more advanced stages of the disease. Despite acknowledging the Will Rogers phenomenon, based on stage migration without a true survival benefit, the available information means that, in most cases, the new stage is assumed, and the indication for available treatments in more advanced stages is extrapolated. This involves making decisions while considering or pursuing the greatest possible benefit for patients, albeit at the cost of potential overtreatment in some cases. It is essential, of course, to discuss the potential benefits and drawbacks with each patient and to make decisions in accordance with their preferences. There are no cost-effectiveness studies available, and, therefore, decisions cannot be based on this criterion. Clinical situations such as this may suggest the need to consider de-escalation treatment strategies.

In patients with advanced disease, the role of NGI techniques should be defined at the time of diagnosis and after treatment with curative intent. In patients with mHSPC, NGI techniques play a role in the assessment of tumor volume and evaluation of systemic therapy vs. targeted therapy within multidisciplinary committees. In non-metastatic CRPC (nmCRPC), NGI should be offered when a change of treatment is being evaluated, and in patients with mCRPC, the use of conventional imaging techniques is strongly recommended.

### 4.2. Challenges

- Implementation of prostate MRI to a greater extent as a routine practice prior to biopsy, taking advantage of all information for T-staging and treatment planning in order to personalize the management of the individual patient.
- Improvement of the diagnostic yield of mpMRI, in particular for the assessment of EPE.
- Reduction in the need for complementary imaging studies for clarifying doubtful findings on conventional techniques, and definition of clinical scenarios, which would benefit from NGI as the initial staging method.
- Assessment of the cost-effectiveness of each imaging technique.
- Definition of the use of NGI in advanced PCa as a predictor of response to salvage radiotherapy (PSMA PET/CT negative), prognostic tool, and evaluation of the response to treatment.
- Absence of prospective studies showing benefits in terms of survival with the use of NGI in high-risk PCa patients.
- There is no evidence of how localized metastatic disease detected by NGI should be treated.

### 4.3. Recommendations

- Explore the possibilities of the combined use of new imaging techniques with emerging tools of artificial intelligence (AI) and virtual reality. Tools such as radiomics may contribute to extracting more objective and quantifiable data from MRI to assist in the diagnosis, assessment of the aggressiveness of tumors, and control of treatment.
- Establish the clinical scenarios in which NGI techniques would be most useful.
- Define the impact of the use of NGI techniques in staging and subsequent change of treatment in terms of outcomes.
- Inclusion of NGI in clinical trials.

## 5. Identification of Patients with Germline Genetic Alterations, Genetic Counselling, and Recommendations for Risk-Adapted Screening

### 5.1. Overview of the Current Situation

In relation to defining PCa patients in whom germline testing would be indicated, the three most used guidelines are those of the European Society of Medical Oncology (ESMO) [51], the 2019 Philadelphia Prostate Cancer Consensus Conference [52], and the National Comprehensive Cancer Network (NCCN^®^) [53] (Table 3).

Genes to be investigated include *BRCA1* and *BRCA2*, *ATM*, *PALB2*, *CHEK2*, *MLH1*, *MSH2*, *MSH6*, *PMS2*, *EPCAM*, and *HOXB13* [53]. According to the ESMO guidelines for screening and early detection, early PSA testing (baseline PSA followed by risk-adaptedfollow-up) can be offered to men >50 years, men >45 years with a family history of prostate cancer, African Americans >45 years, and *BRCA1/2* carriers >40 years [51]. Germline *BRCA1/2* mutations confer a lifetime 33% cumulative risk of PCa. For *BRCA1* mutation carriers, an estimated relative risk (RR) of 1.8–3.75-fold by age 65 years has been estimated [54], and for *BRCA2* mutation carriers, a RR of 2.5–4.6, increasing to a RR of 8–23 in men younger than 55 years. Additionally, *BRCA1/2* mutations have been reported to result in an aggressive pattern of disease (Gleason score ≥ 8 and more advanced stages) [55,56].

Moreover, the presence of *BRCA1* and *BRCA2* mutations in PCa patients increased the risk of developing other tumors, such as male breast cancer, cancer of the pancreas, and melanoma, as compared with the incidence in the general population. Screening guidelines for these tumors have also been recommended by the NCCN^®^ (Table 4).

Targeted screening in men with a family history of PCa has been the focus of interesting studies. The PROFILE cross-sectional pilot study was designed to assess the feasibility and safety of PCa screening using ultrasound-guided transrectal prostate gland biopsy with or without diffusion-weighted MRI (DW-MRI) [57]. The study also assessed the usefulness of collecting data on levels of PSA, analysis of profiles of single-nucleotide polymorphisms (SNPs), and DW-MRI as screening tests in men with a family history of PCa [57]. The study population included 100 patients, with a diagnosis of PCa in 25 and csPCa in 12 (48%). Among the 25 patients with PCa, the PSA level was >3 ng/mL in 13 (52%), and these patients would most likely not have undergone biopsy on the basis of the traditional PSA-based screening schedules [57]. Additionally, novel urine tests such as the MiPS (Mi-Prostate Score) or SelectMDx^®^ can be useful in the PCa screening process in patients with germline mutations [58].

### 5.2. Challenges

- Identify patients with genetic predisposition to PCa in the local setting.
- Indications of screening procedures in healthy subjects with a history of PCa.
- Definition of genes of interest and pathogenic variants for genetic testing.
- Timing of referral for genetic testing.
- Establish the schedule of PSA testing at 40 years and additional screening procedures.
- Establish the necessary follow-up schedule for the early diagnosis of other related tumors.
- Determine the follow-up schedule according to the results of genetic testing.

### 5.3. Recommendations

- Proposal of genetic testing in hereditary cancer predisposition: metastatic PCa Gleason score ≥ 7; PCa with Gleason score ≥ 7 at age < 55 years; PCa with Gleason score ≥ 7 and family history of breast and/or ovarian cancer, or 2 or more cases of PCa in the same family branch; PCa < 55 years and family history of 2 or more cases of PCa, or hereditary breast and ovarian cancer; and PCa with cribriform histological pattern (ductal or intraductal).
- If genetic testing on healthy tissue extracted from a paraffin block of the patient diagnosed with PCa (ideal index case) is not possible, a healthy family member may be considered:
  – A healthy first-degree relative of an individual who meets high-risk criteria for hereditary breast and ovarian cancer syndrome and has a >10% probability of identifying a mutation according to risk estimation models.
  – Evaluation by a committee of hereditary cancer of the following cases: healthy subject with a first-degree relative with colorectal cancer <50 years old and where tumor study is not possible; healthy first-degree relative of a patient with high-grade epithelial ovarian cancer; healthy subject with a first-degree relative with breast cancer meeting criteria for genetic study; healthy first-degree relative of a patient and family meeting criteria for hereditary PCa; and healthy first-degree relative of a patient and family meeting criteria for hereditary pancreatic cancer.
- The genetic panel should include *BRCA1*, *BRCA2*, *MLH1*, *MSH2*, *MSH6*, *HOXB13* (*G84E* variant), *ATM*, *CHEK2*, *PALB2*.

## 6. Clinical Application and Molecular and Genetic Characterization of the Disease

### 6.1. Overview of the Current Situation

In order to make a better selection of targeted treatment options, molecular and genetic profiles have been introduced for subtyping solid cancer tumors, including PCa. In molecular taxonomy analysis of 333 primary PCa tumors in the framework of The Cancer Genome Atlas (TCGA), it was found that 74% of these tumors corresponded to one of seven subtypes defined by specific gene fusions (*ERG*, *ETV1/4*, *FLI1*) or mutations (*SPOP*, *FOXA1*, *IDH1*) [59]. Moreover, a presumed actionable lesion in the PI3K or MAPK signaling pathways was documented in 25% of these tumors, and inactivated DNA repair genes in 19%. This integrative evaluation of PCa tumors demonstrated molecular heterogeneity and recognized new alterations and diversity of subtypes [59].

An analysis of the genomic landscape based on whole exome and transcriptome sequencing of bone or soft tissue tumor biopsies from a cohort of 150 patients with mCRPC showed that 89% of them harbored a clinically actionable aberration [60]. In aggregate, 71.3% of cases harbored *AR* pathway aberrations, 49% alterations of the PI3K pathway, 22.7% alterations of the DNA repair/recombination genes, 21% alterations in the cell cycle pathway, and 18% alterations in the Wnt signaling pathway. These multiple new discoveries within the advanced stage of PCa that have not been previously observed in primary tumor profiling could impact treatment decisions in a relevant number of affected PCa patients [60].

Molecular biomarkers have been developed to accurately stratify risk in PCa patients, but evidence supporting the adoption of genomic classifiers in clinical practice is still inconsistent. Jairath et al. [61] conducted a systematic review of the literature to assess the utility of the Decipher (22-gene genomic classifier [GC]), summarizing data reported in 42 studies with 30,407 patients (localized, prostatectomy, nmCRPC, mHSPC). In 24 studies, the GC was a prognostic factor for all endpoints (biochemical failure, metastasis, and cancer-specific and overall survival) on multivariate analysis, whereas in 5 studies, the use of GC changed the management in active surveillance and post-prostatectomy. The authors conclude that the Decipher genomic classification tool helps identify which cancers are more or less aggressive, which in turn aids in personalized treatment decision-making [61]. However, negative results were obtained using the 17-gene Oncotype DX Genomic Prostate Score (GPS) as a predictor of outcomes in a multicenter active surveillance cohort [62]. Adverse pathology (Gleason Grade Group [GG] ≥ 3 and ≥pT3) after initial surveillance in patients treated with radical prostatectomy was the primary study endpoint. It was found that risk stratification for adverse pathology did not improve by the addition of GPS to a model containing PSAD and GG as compared with clinical variables alone [62].

Finally, a recent study evaluated the ability of the clinical cell risk score (CRS), which combines the University of California, San Francisco’s Cancer of the Prostate Risk Assessment (CAPRA) and the cell cycle progression (CCP) molecular score, for predicting the risk of metastasis in men receiving dose-escalated radiation therapy (RT) with or without androgen deprivation therapy (ADT) [63]. All three scores, CRS, CAPRA, and CCP, were significant predictors of metastasis with HR of 2.22, 1.39, and 2.03, respectively, and adding clinically actionable information relative to guideline-recommended treatments [63].

**Figure 3:**
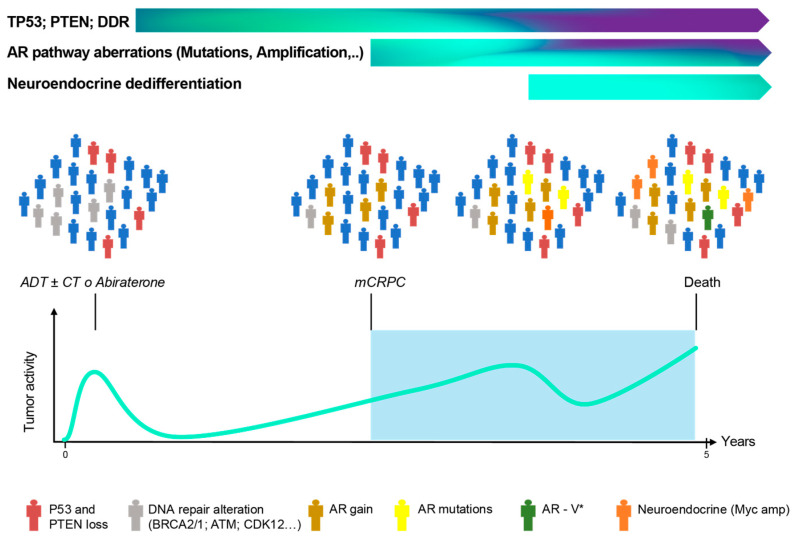
Molecular biomarkers in advanced PCa. * Any or all splice variants of the androgen receptor. The most common is AR-V7, and other AR-Variants include ARV-1, 3, or 5.

PCa is a dynamic disease, and the course of the disease should be considered at the time of identifying the most adequate molecular biomarkers (Figure 3).

In relation to molecular subtyping classification to identify clinically distinct subgroups that benefit from specific therapies, Zhao et al. [64] used the PAM50 gene expression classifier to differentiate prostate cancer samples into luminal- and basal-like subtypes in a retrospective and a prospective cohort. The PAM50 classifier consistently segregated PCa into 3 subtypes in both cohorts: luminal A and B, and basal. Luminal-like cancers showed an enrichment of luminal lineage markers (e.g., NKX3.1 and KRT18), whereas basal-like cancers showed enrichment of the basal lineage CD49f signature. Luminal A and luminal B subtypes showed an association with increased expression and signaling of the androgen receptor, but a significant postoperative response to ADT was only observed in the luminal B subtype. Therefore, this gene expression classifier has a potential clinical value for predicting response to ADT after surgery, contributing to personalizing ADT therapy [64]. Hamid et al. [65] evaluated the predictive value of the PAM50 classifier in men with mHSPC and high-risk features in 168 pre-ADT specimens. In the ADT arm, luminal B subtype was associated with shorter overall survival vs. basal (HR 1.75, *p* = 0.05), whereas patients with luminal B subtype treated with ADT + docetaxel showed significant improvement in time to castration resistance and overall survival. The study demonstrates that gene expression profiling identified tumor subtypes associated with differential benefit from chemo-hormonal therapy for mHSPC [65].

Tumors with gene alteration that affect homologous recombination repair (such as *BRCA1* and *BRCA2*) are sensitive to poly(adenosine diphosphate–ribose) polymerase (PARP) inhibitors. The PROFOUND prospective biomarker-selected, phase 3 trial, enrolled patients with mCRPC with at least one alteration in the *BRCA1*, *BRCA2,* or *ATM* (Cohort A) whose disease had progressed during previous treatment with a next-generation hormonal agent [11]. The study also included Cohort B, comprising patients with alterations in other genes involved in homologous recombination repair [11]. The duration of overall survival was longer in patients treated initially with olaparib than in those assigned to control treatment (enzalutamide or abiraterone plus prednisone). Also, patients with tumors harboring *BRCA1* or *BRCA2* alterations appeared to obtain the greatest benefit in terms of overall survival from treatment with olaparib [11].

In the PROpel randomized, double-blind phase 3 trial of first-line therapy for patients with mCRPC, olaparib + abiraterone showed a consistent trend toward better overall survival versus abiraterone + placebo, with the greatest benefit in the group of mutations of the *BRCA* gene [66]. In the TALAPRO-2 phase 3 study, the combination of the PARP inhibitor talazoparib plus enzalutamide vs. placebo and enzalutamide in mCRPC reduced the risk of radiographic progression–free survival (rPFS) in the overall study population by 55% and in all subgroups of homologous recombination repair (HHR) gene-deficient patients enrolled in the study, most notably in patients with *BRCA* alterations [67,68]. Patients with BRCA alterations experienced an 80% reduction in rPFS compared with non–*BRCA*-mutated patients (*p* < 0.001). Also, patients on the combination arm with *BRCA* alterations had a 32% reduction in rPFS vs. patients with other mismatch-repair gene alterations on the combination arm [68]. The clinical benefit of treatment combination in tumor HRR gene alterations will be clarified in additional long-term safety and follow-up analysis.

Finally, biomarkers are difficult to realistically adopt since validation studies are lacking. The prognostic value of PTEN-Rb1-p53 has been proposed; PTEN has also been suggested as a potential predictor of benefit from docetaxel in the mHSPC. In addition, genomic platforms, specifically PAM50 and Decipher, have also been proposed as predictors of benefit from docetaxel in mHSPC. However, none of these proposals have yet been definitively validated. Interestingly, in a retrospective study of 1,561,203 patients with a PCa diagnosis collected between 2011 and 2021 from the PearlDiver™ Mariner database, tissue-based genetic testing was used in less than 2% of PCa patients, whether at initial diagnosis or after surgical treatment [69]. The authors concluded that more scientific evidence is needed to elucidate the role of genomic tests in supporting PCa treatment decisions [69].

### 6.2. Challenges

- Identification and validation of molecular biomarkers predictive of response to treatments in localized disease, as well as in more advanced stages.
- Need to better understand the benefit of PARP inhibitors (PARPi) + androgen receptor signaling pathway inhibitors (ARPi) to define the context where results can be optimized.
- Assessment of benefits/toxicity of concomitant and sequential treatment with PARPi and ARPi.
- Mutations in the androgen receptor gene. Pharmacological development of ARV-110 and ODM 201 as promising therapies.
- Role of microsatellite instability (MSI) and other molecular biomarkers in the development of immunomodulatory drugs.
- Awareness of the importance of molecular characterization of PCa.
- Knowledge of the main altered pathways and specific training.
- Implementation of molecular biomarkers in the design of clinical trials.

### 6.3. Recommendations

- Critical analysis of published studies with general recommendations and the possibility of their implementation in national hospital settings in different clinical scenarios.
- Facilitate access to molecular characterization of PCa and implementation of specific training.
- Appropriate development of molecular biomarkers.
- Participate in the design of clinical trials with the implementation of molecular biomarkers in both localized and advanced disease.
- Potentiation of translational research programs.
- Participation in clinical trials and multicenter studies of molecular biomarkers.

In summary, a Spanish panel of PCa experts participated in the ENFOCA2 project aimed to provide guidance for clinicians on current relevant aspects related to screening, risk-based diagnosis, selection of novel imaging techniques, molecular biomarkers, and indications of genetic testing. Clinicians need to have updated information on advances in the different aspects of PCa to implement them in clinical practice and thereby provide optimal health care to their patients.

## Figures and Tables

**Table 1:** Components of an integral perspective in the management of patients with PCa.

**Diagnosis, staging and treatment of PCa requires** **an integral approach including:** – Diagnosis (clinical, laboratory, imaging, biopsies, histopathological) – Surgical, radiotherapy, medical and supportive treatment – Hereditary/family studies – Psychological, emotional and social support – Treatment of sequelae and iatrogenesis – Supportive and palliative care	**Specialists and professionals involved:**	
	- Radiology - Pathology - Nuclear Medicine - Urology - Radiation Oncology - Medical Oncology - Hospital Pharmacy	- Clinical Research Unit - Internal Medicine - Oncogeriatrics - Specialized Nursing - Palliative Care - Social workers - Administrative staff
**Integral approach requires multidisciplinary approach:** – Team of specialists and professionals cooperating (not competing) – Interchange of ideas and proposals regularly	**Possible models:** A responsible physician and consultation with other specialists if considered appropriate Multidisciplinary tumor committee Functional Units Units of practice and integrated management Monographic institutes and hospitals	
**To answer patient-focused questions:** – What does this patient need? – What each specialist from the MDT can offer to the patient? – Which should be the current global strategy and in the long term? – Who is the most adequate for the execution of each task? – In what order?		
**There are still many PCa patients who do not receive an integral and multidisciplinary approach oriented to their current needs and to their global strategy.**		

**Table 2:** Guidelines of the EAU for PCa screening in the general population.

Recommendation	Strength Rating of the Evidence
– Do not subject men to prostate-specific antigen (PSA) testing without counselling them on the potential risks and benefits	Strong
– Offer an individualized risk-adapted strategy for early detection to a well-informed man with a life-expectancy of at least fifteen years.	Weak
– Offer early PSA testing to well-informed men at elevated risk of having PCa: Men from 50 years of age Men from 45 years of age and a family history of PCa Men of African descent from 45 years of age Men carrying *BRCA2* mutations from 40 years of age	Strong
– Offer a risk-adapted strategy (based on initial PSA level), with follow-up intervals of two years for those initially at risk: Men with a PSA level of < 1 ng/mL at 40 years of age Men with a PSA level of < 2 ng/mL at 60 years of age Postpone follow-up up to eight years in those not at risk.	Weak
– Stop early diagnosis of PCa based on life expectancy and performance status; men who have a life-expectancy of <fifteen years are unlikely to benefit	Strong
– In asymptomatic men with PSA level between 3 and 10 ng/mL and a normal DRE, one of the following tools for biopsy indication: Risk-calculator correctly calibrated to the population prevalence MRI of the prostate	Strong

Reference [26].

**Table 3:** Germline testing recommendations.

ESMO [51]	Philadelphia Consensus [52]	NCCN^®^ [53]
Metastatic disease: – In castration-resistance PCa patients Localized disease – ≥2 first-degree relatives of the same family side diagnosed with hereditary cancer predisposition (breast, ovary, prostate, pancreas, melanoma, sarcoma, adrenal, brain, colon, endometrium, gastric, thyroid, renal) Pathogenic mutations in hereditary cancer risk genes identified by tumor testing: – Refer to a hereditary cancer unit for assessing germline testing	Healthy men with cancer family history: – Parent or sibling or second-degree relative with at least 1 on the following criteria: Diagnosed with PCa < 60 years Death due to PCa or Alive with metastatic disease – 2 or more cases with hereditary breast and ovarian cancer tumors or Lynch syndrome (especially <50 years)	– By prostate cancer stage or risk group (diagnosed at any age) Metastatic, regional (node positive), very-high-risk or high-risk localized – By family history and/or ancestry * – By prostate cancer tumor characteristics (diagnosed at any age) Intermediate-risk PCa with intraductal/cribriform histology – By prostate cancer AND a prior personal history of any of the following cancers: Exocrine pancreas, colorectal, gastric, melanoma, upper tract urogenital, glioblastoma, biliary tract, and small intestine

* ≥1 first-, second-, or third-degree relative with: breast cancer at age ≤ 50 and colorectal or endometrial cancer at age ≤ 50 and male (sex assigned at birth) breast cancer at any age; ovarian cancer at any age; exocrine pancreatic cancer at any age; metastatic, regional (node positive), very-high-risk or high-risk PCa at any age. ≥1 first-degree relative (parent or sibling) with PCa at age ≤ 60. ≥2 first-, second-, or third-degree relative with breast cancer at any age or prostate cancer at any age. ≥2 first-, second-, or third-degree relative with Lynch syndrome-related cancers, especially if diagnosed at <50 and colorectal, endometrial, gastric, ovarian, exocrine pancreas, upper tract urothelial, glioblastoma, biliary tract, and small intestine cancer. A known family history of familial cancer risk mutation (pathogenetic/likely pathogenic variants), especially in *BRCA1*, *BRCA2*, *ATM*, *PALB2*, *CHEK2*, *MLH1*, *MSH2*, *MSH6*, *PMS2*, and *EPCAM*. Ashkenazi Jewish ancestry.

**Table 4:** Risks of cancer tumors other than prostate cancer in *BRCA1* and *BRCA2* carriers.

Tumors	*BRCA1* Carriers	*BRCA2* Carriers	General Population	Screening Per 2021 NCCN^®^ Guidelines
Male breast cancer	7–8%	1%	0.1%	Breast self-examination education at 35 years Clinical breast examination at 35 years Consider annual mammography if gynecomastia
Pancreas	5–7%	2–3%	~1.5%	Can consider screening (MRI/MRCP/EUS) ideally in research setting
Melanoma	5% ?	?	~2.5% (Whites) ~0.1% (Blacks)	Reasonable to do annual examination and UV light protection

MRI: magnetic resonance imaging; MRCP: magnetic resonance cholangiopancreatography; EUS: endoscopic ultrasound; UV: ultraviolet. ?: not confirmed/unknown.

## Data Availability

No new data were created or analyzed in this study. Data sharing is not applicable to this article. Data from this publication have previously been shared at a National SOGUG Symposia in November 2024.
